# Eyelash length attractiveness across ethnicities

**DOI:** 10.1038/s41598-023-41739-5

**Published:** 2023-09-08

**Authors:** Farid Pazhoohi, Alan Kingstone

**Affiliations:** 1 School of Psychology, University of Plymouth, Drake Circus, Plymouth, PL4 8AA UK; 2 Present Address: Department of Psychology, University of British Columbia, 2136 West Mall, Vancouver, V6T 1Z4 Canada

**Keywords:** Human behaviour, Biological anthropology

## Abstract

Eyelashes evolved to protect eyes. An optimum eyelash length functions to protect eyes from external hazards such as contaminations, excessive evaporation or shear stress from airflow. They can also be an indicator of a person's health as various congenital and noncongenital diseases can lead to short or long eyelashes. The current study aimed to extend a recent investigation on the preference for eyelash length in humans from an evolutionary adaptive perspective. Specifically, the current study tested whether the inverted-U function for eyelash length preference recently reported for White faces, generalises to other ethnicities, and whether ethnic background modulates preference for eyelash lengths. To investigate this question, men and women of Asian, Black, and White ethnicities from the U.S. rated the attractiveness of female Indian, Asian, Black, and White faces with varying eyelash lengths. The eyelashes ranged in length from no eyelashes to half the width of an eye. Results showed that Asian, Black, and White men and women preference for eyelash length followed an inverted-U function across all four ethnicities, supporting a general preference for human eyelash length that is approximately one-third the width of an eye. In addition, the results showed that the most attractive eyelashes for Black women were skewed toward a greater eyelash-length to eye-width ratio when compared to the other images. The source of this skew is presently unknown, as it could reflect a change in perceptual sensitivity to eyelash length with skin colour or changes in preference related to perceptions of participants’ ethnicity.

## Introduction

Pazhoohi and Kingstone^1^ introduced the possibility that the perception of eyelash length attractiveness might dovetail with the proposed phylogenetically adaptive functionality of eyelash length^2^. Specifically, arguing from an adaptive evolutionary perspective that attractiveness is an honest signal of health and genetic quality^3,4^, Pazhoohi and Kingstone^1^ proposed that eyelash length should be perceived as most attractive at the length where: (a) it has the highest functionality in minimizing evaporation and contamination at the ocular surface^2^, and (b) it is associated with the highest health condition of the eyelashes^5,6^.

Amador et al.^2^ measured eyelash length and eye width of twenty-one mammals and found that eyelash length is phylogenetically constant and about one-third of eye width (L/W ratio of 0.35 ± 0.15). Using simulated eyelashes and theoretical models they also showed that such an eyelash length to eye width ratio is optimum for minimizing evaporation and contamination at the ocular surface, yet avoiding the impediment of the vision^2^.

From a health and genetic point of view, previous research has shown that long eyelashes^6,7^ and short eyelashes^5,8^ can serve as indicators of congenital and noncongenital diseases. Accordingly, Pazhoohi and Kingstone^1^ hypothesized that face attractiveness would show an inverted-U relationship with the eyelash/eye L/W ratio; with the highest attractiveness arising around the 0.35 L/W ratio. Participants were asked to rank the attractiveness of both a man's face and a woman's face. These faces varied in the length of their eyelashes, ranging from no eyelashes (0 L/W) to very long eyelashes (0.5 L/W). The results showed that the preference for eyelash length follows an inverted-U function dovetailing with the proposed evolutionary adaptive length of eyelashes. Interestingly, the highest ratings for male faces skewed toward a shorter ratio than female faces. Also, while very short or no eyelashes were ranked as least attractive for women faces, very long eyelashes were ranked as the least appealing for men faces. This discrepancy in eyelash preference between male and female faces was attributed to factors such as cultural norms^1^.

A natural question following this finding is whether faces of different skin colour, which can suggest differences in ethnicity modulate the preferences for the eyelashes. Cross-cultural research has shown differences in attractiveness preferences in facial features such as shape, lips proportion, skin color, and dimorphic traits^9–13^, which potentially are a result of adaptations under different environmental conditions^14–16^. These studies have shown that facial features might be regarded differently and preferred by one's own-ethnicity compared to other ethnicities^10,17^. Therefore, the findings by Pazhoohi and Kingstone^1^ might not generalize across different ethnicities. In other words, it is crucial to examine whether the previous finding with an inverted-U pattern^1^ will be generalized and observed across diverse ethnicities. The current research explored this question, and considered the self-identified ethnicity of the observer as well. In other words, the aim of the current study is to extend the findings of Pazhoohi and Kingstone^1^ by testing the inverted-U function of preference for eyelash length across stimuli and participants differing in ethnic backgrounds. In addition, the current study extends the previous study methodologically. Pazhoohi and Kingstone^1^ asked participants to rank the images collectively based on their relative level of attractiveness. In the present study participants rated each image individually on its perceived attractiveness. Finally, the current study tested for the presence of sex differences in the perception of attractiveness as a function of eyelash length and ethnicity. Previous research has reported sex differences in how the brain processes faces in general and facial attractiveness in particular [e.g., Refs.^18,19^; for reviews see Refs.^20,21^]. There is also evidence that men and women preferentially attend to different facial features^22^ resulting in different regions of the face being prioritized^23^. Moreover, recent research has indicated the sex differences and ethnicity may interact^24,25^, making it a relevant question to investigate the effects of these two variables on the perception of eyelashes.

## Methods

### Participants

A total of 319 individuals (142 men and 177 women) aged between 21 and 74 years (*M* = 41.03, SD = 11.82) participated and completed an online survey. Participants were recruited from the U.S. MTurk and self-identified as Asian (N = 100), Black (N = 99), and White (N = 120). The choice of words for ethnicities in this paper is following MTurk’s choices, i.e., “Asian”, “Black/African American”, and “White/Caucasian”. A total of 125 participants (39.2%) reported being married, and 19.4% reported being not married but in a relationship. Additionally, 32.3% reported being single, and 9.1% were either widowed, divorced, or separated. As for their highest educational degree, two reported elementary school, 23.5% had a high school diploma, 11.0% had a post-secondary diploma, 49.5% had an undergraduate degree, and 15.4% had a postgraduate degree. All participants provided informed consent to take part in the study. None of the participants were excluded from the analysis.

### Stimuli and procedure

**Figure 1:**
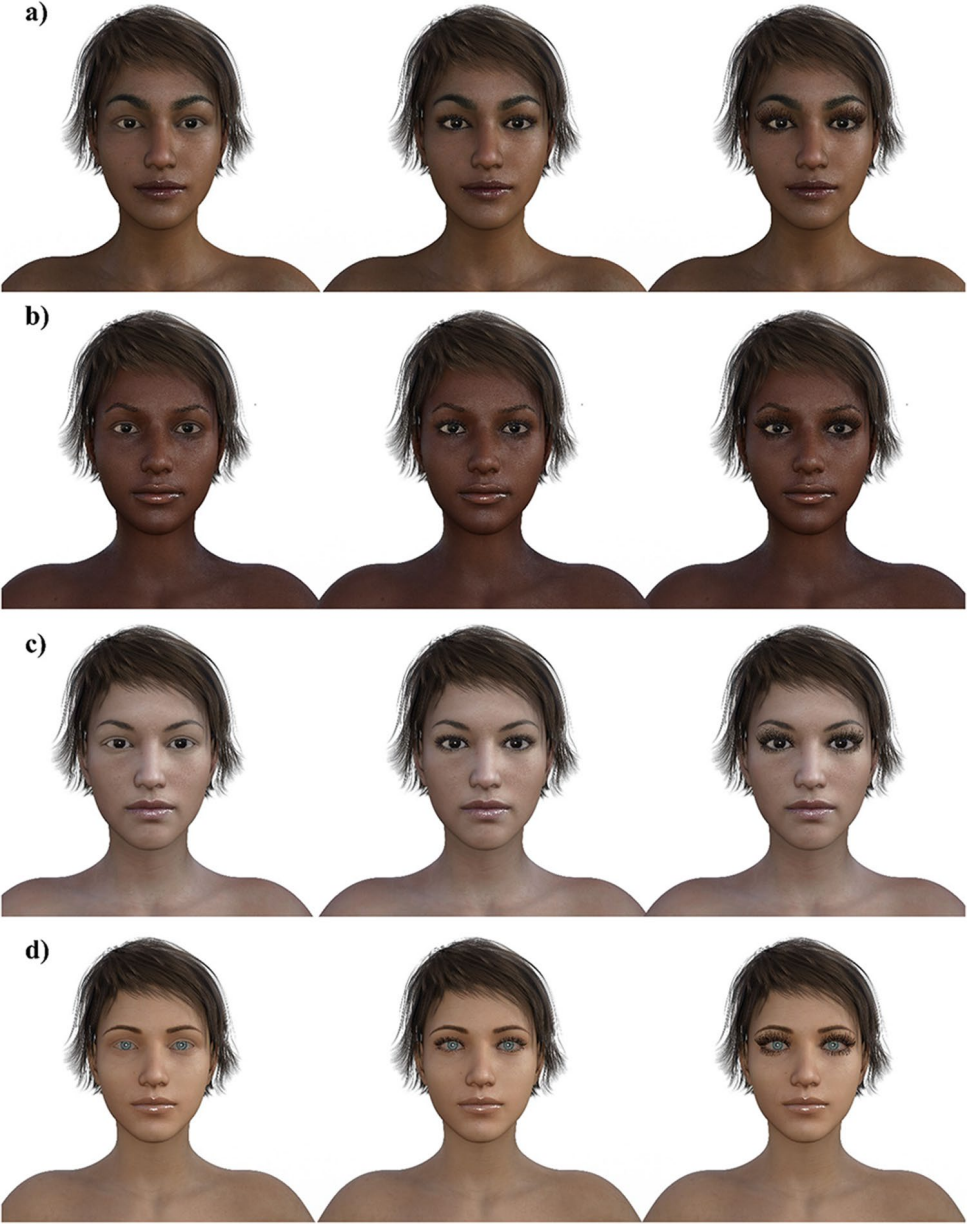
Examples (**a**) Indian, (**b**) Black, (**c**) Asian, and (**d**) White faces varying in eyelash length to eye width ratio; left images 0, middle images 0.25, and right images 0.50 ratios.

Four female facial images differing in ethnicity were generated using Daz3D software (‘Beauties of the world’ package was used to create the ethnicities from different broad regions. They are identified by the package as faces from Northeast Asia, West Africa, South Asia, and Northern Europe. Accordingly, we refer to each group of stimuli in our paper as Asian, Black, Indian and White, respectively. All the facial images were of the same identity.). Eleven copies of each ethnicity (Asian, Black, Indian (South Asian), and White) varying in eyelash length were created using Photoshop (see Fig. 1 for examples). A separate eyelash was created for each length and was pasted on the faces without eyelashes (length zero). A Photoshop artist created the eyelashes. To mimic the natural appearance of the eyelashes, they were drawn shorter on the lower lid and the inner eye. The length of the eyelash at the center of the upper lid was taken as the length of the eyelash. For each stimulus ethnicity, eleven eyelash-length to eye-width ratios were created ranging from 0 to 0.5, incrementing in 0.05. Each stimulus ethnicity was presented to the participants in a separate block, and the order of the blocks as well as the order of stimuli within each block were randomized. In Pazhoohi and Kingstone^1^ female eyelash lengths were manipulated for white faces with brown eyes. To explore if eye color interacts uniquely with eyelash length, blue eyes were used for the white-faced stimuli in the present studies. The results fail to suggest any unique interaction. The blue-eyed white face eyelash length results replicate the inverted U pattern obtained for brown eyed white faces in Pazhoohi and Kingstone^1^; and they are not unique from the other faces in the present study.

After consenting to participate and answering the demographic questions, the participants were instructed that they would see faces with different eyelash lengths, and their task was to rate the attractiveness of the faces as a function of the different eyelash lengths. They answered the question “How attractive do you think this woman is?” on a 7-point numerical scale from 1 (not at all) to 7 (extremely). In total, participants completed 44 trials (4 ethnicity blocks × 11 eyelash lengths per block). The rating task was self-paced.

### Ethics declarations

All participants provided informed consent to take part in the study. This research was approved by the Behavioural Research Ethics Committee of the University of British Columbia and was conducted in accordance with the Declaration of Helsinki as it pertains to research with human participants.

## Results

A 2 (Participant Sex) × 3 (Participant Ethnicity: Asian, Black, and White) × 4 (Stimulus Ethnicity: Asian, Black, Indian, and White) × 11 (Eyelash Length) mixed ANOVA was performed with Participant Sex and Participant Ethnicity as a between-subjects variables, and Stimulus Ethnicity and Eyelash Length as within-subjects variables on attractiveness ratings. All post hoc comparisons reported here, and throughout the results, applied Bonferroni correction and is reflected in the *p* values. Table 1 shows the results for the main and interaction effects.

**Table 1:** Mixed ANOVA main and interaction effects results for eyelash attractiveness ratings as a function of participant sex, participant ethnicity, stimulus ethnicity, and eyelash length.

Predictor	*df*_*num*_	*df*_*den*_	*F*	*p*	Partial* ŋ*^2^
Between-subjects effects					
Participant sex	1	313	0.01	0.917	0.00
Participant ethnicity	2	313	0.46	0.631	0.01
Participant sex × participant ethnicity	2	313	0.87	0.418	0.01
Within-subjects effects					
Stimulus ethnicity	3	939	21.16	< 0.001	0.01
Stimulus ethnicity × participant sex	3	939	3.05	0.028	0.01
Stimulus ethnicity × participant ethnicity	6	939	0.73	0.625	0.01
Stimulus ethnicity × participant sex × participant ethnicity	6	939	0.70	0.651	0.01
Eyelash	10	3130	166.02	< 0.001	0.35
Eyelash × participant sex	10	3130	2.87	0.001	0.01
Eyelash × participant ethnicity	20	3130	2.11	0.003	0.02
Eyelash × participant sex × participant ethnicity	20	3130	0.94	0.542	0.01
Stimulus ethnicity × eyelash	30	9390	26.93	< 0.001	0.08
Stimulus ethnicity × eyelash × participant sex	30	9390	1.28	0.139	0.01
Stimulus ethnicity × eyelash × participant ethnicity	60	9390	1.53	0.005	0.01
Stimulus ethnicity × eyelash × participant sex × participant ethnicity	60	9390	1.02	0.435	0.01

Results for the main effects of Stimulus Ethnicity and Eyelash Length were significant, and both were qualified by significant Stimulus Ethnicity × Participant Sex and Eyelash Length × Participant Sex two-way interactions. Women rated Indian ethnicity (*M* = 4.10, *SEM* = 0.08, 95% CI [3.93, 4.25]) more attractive than Black (*M* = 3.79, *SEM* = 0.09, 95% CI [3.62, 3.96], *p* < 0.001) and White ethnicities (*M* = 3.61, *SEM* = 0.08, 95% CI [3.45, 3.78], *p* < 0.001); Women also rated Asian ethnicity (*M* = 3.95, *SEM* = 0.07, 95% CI [3.80, 4.11]) more attractive than White ethnicity (*p* < 0.001). Men rated Indian (*M* = 3.96, *SEM* = 0.09, 95% CI [3.78, 4.14], *p* = 0.001) and Asian (*M* = 4.01, *SEM* = 0.08, 95% CI [3.83, 4.18], *p* < 0.001) more attractive than Black ethnicity (*M* = 3.68, *SEM* = 0.10, 95% CI [4.48, 3.88]). Also, men rated Asian ethnicity more attractive than White ethnicity (*M* = 3.75, *SEM* = 0.09, 95% CI [3.57, 3.94], *p* = 0.005). As for the significant Eyelash Length × Participant Sex interaction, no sex difference was found in the post-hoc analysis between men and women in ratings of each eyelash ratio (all *p*s > 0.061).

**Figure 2:**
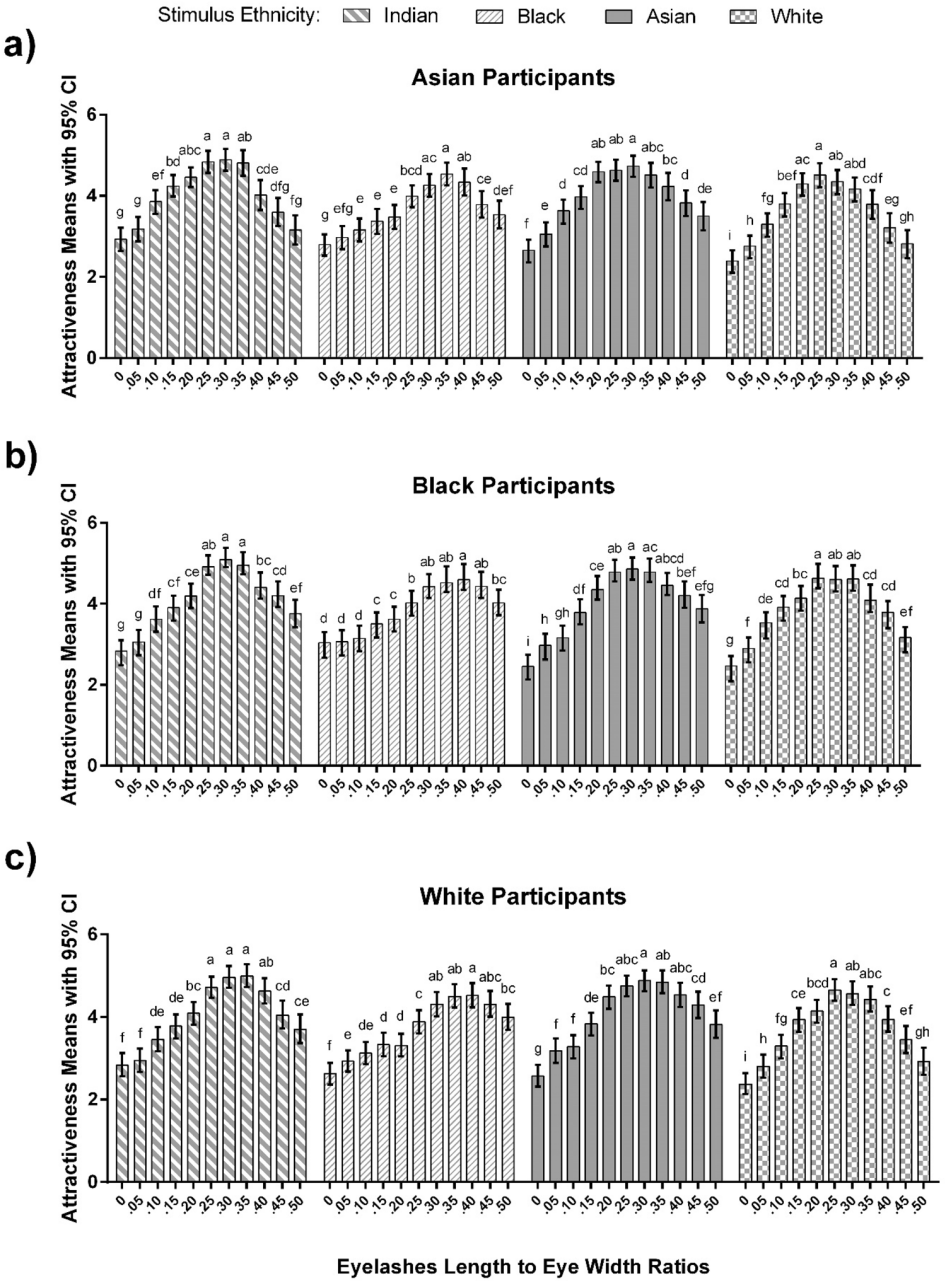
(**a**) Ratings of attractiveness (means ± 95% CI) for Asian participants rating Indian, Black, Asian and White female faces with different eyelash length to eye width ratios (L/W), (**b**) Ratings of attractiveness (means ± 95% ci) for black participants rating Indian, Black, Asian and White female faces with different L/W ratios, and (**c**) Ratings of attractiveness (means ± 95% ci) for white participants rating Indian, Black, Asian and White female faces with different L/W ratios. Means not sharing the same letters are significantly different (*p* < 0.01). For example, for Asian participants rating Indian faces, ratios 0.20, 0.25, 0.30, and 0.35 are indicated by the letter ‘a’, meaning they are not significantly different from each other. Similarly, ratios 0.15, 0.20, and 0.35, share letter ‘b’ meaning they are not significantly different from each other. However, ratios 0.25 and 0.30 are different from 0.15, as they do not share a similar letter.

Eyelash Length × Participant Ethnicity and Stimulus Ethnicity × Eyelash Length two-way interactions were qualified by a significant three-way Stimulus Ethnicity × Eyelash Length × Participant Ethnicity (see Table 1 for details). Pairwise comparisons indicated that Asian participants rated eyelashes of Indian, Asian, and White women with 0.20, 0.25, 0.30, and 0.35 eyelash/eye ratios as the most attractive than the other ratios, while they rated the ratios of 0.30, 0.35, and 0.40 for Black women as the most attractive (see Fig. 2a).

For Black participants, the ratios of 0.25, 0.30, and 0.35 on Indian and White women faces were the most attractive compared to other ratios, and 0.25, 0.30, 0.35, and 0.40 for Asian women faces were rated as highest. Black participants, similar to Asian participants rated the ratios of 0.30, 0.35, and 0.40 for Black women as the most attractive eyelashes (see Fig. 2b).

The pairwise comparisons showed that White participants rated 0.25, 0.30, and 0.35 eyelash/eye ratios as the most attractive for Indian and White women, and rated 0.25, 0.30, 0.35, and 0.40 ratios for Asian as the most attractive ratios. White participants rated ratios of 0.30, 0.35, 0.40, and 0.45 for Black women as the most attractive (see Fig. 2c).

Results also showed that no eyelash and very short eyelash lengths were rated the least attractive for all Asian, Black, and White participants ratings for all the ethnicities of Indian, Black, Asian and White women. Figure 2 details the comparisons between each eyelash/eye ratios.

In summary, there were commonalities and differences between the groups of participants in terms of their preferences. All participants preferred all faces in the 0.30–35 range. All participants also preferred Asian, White and Indian faces at the 0.25 ratio, and Black faces at the 0.40 ratio. In terms of differences, Asian participants were unique in preferring faces (save for Black faces) at the 0.20 ratio; whereas only Black and White participants preferred Asian faces at the 0.40 ratio; and only White participants preferred Black faces at the 0.45 ratio.

To confirm the inverted-U function for the ratings, a series of quadratic regressions were conducted and significant relationships were found between eyelash length and attractiveness ratings for each stimulus ethnicity by participant ethnicity (Supplementary Table S1).

## Discussion

Pazhoohi and Kingstone^1^ recently discovered that as eyelashes lengthen, faces are perceived to be more attractive. But only up to a point, beyond which the perceived attractiveness of faces begins to fall with increasing eyelash lengths. In the original work that yielded this inverted-U function, the female faces that were rated for their attractiveness were white skinned. In the present study we asked if the inverted-U function occurs for faces of other skin colors/ethnicities. And if perception varies systematically for observers of different ethnicities.

The results showed that all Asian, Black and White participants rated different ethnicities of Indian, Asian, Black, and White female faces in a similar fashion, regardless of eye colour, yielding an inverted-U relationship. This suggests a general inverted-U function for the perceived attractiveness of faces that mirrors the optimal length for eyelashes in terms of their evolutionary development—balancing eye protection with vision. Amador et al.^2^ identify the optimal eyelash length at about one-third the eye width (L/W ratio of 0.35 ± 0.15). Our results are convergent with this ratio. They also suggest, however, some variation across the ethnicities on the most preferred ratios. In the present study the preferred L/W ratio ranged between 0.25 to 0.40 with slight differences emerging as a function of an observer's ethnicity. For example, Asian participants rated Asian faces as the most attractive for ratios 0.20–0.35, while Black participants preferred Asian faces for ratios 0.25–0.40.

A difference for the highest attractive ratios were also found to vary as a function of the image itself. Results showed that ratings of Black women's eyelashes by all Asian, Black and White participants were slightly skewed toward a larger ratio than the other faces (see Fig. 2). Previous research has indicated differences in perception of attractiveness as a function of skin color^26,27^, and facial racial physiognomy and characteristics^11,27,28^. While the source of this skew is presently unknown, it could reflect a change in perceptual sensitivity to eyelash length with skin colour. In other words, the slight difference found in the most attractive eyelash length among ethnicities in this study might be due to the color contrast between black eyelashes and skin color of ethnicities, with Black faces providing less contrast. Nonetheless, it might have been expected that individuals would rate the most attractive eyelash length for Indian faces higher compared to White and Asian faces, as they would provide less contrast with the skin color. However, this was not the case, which raises the need for future research to combine incremental skin color variations from light to dark with different eyelash lengths to examine their impact on the perception of attractiveness.

As for the least attractive eyelash length, the results dovetail with those of Pazhoohi and Kingstone^1^, where female faces with no eyelashes and very short eyelashes were the perceived to be the least attractive. Similarly, all ethnicities rated faces of every ethnicity with no and very short eyelash length as the least attractive compared to the other eyelash lengths.

The results of this study also showed that men and women perceive the effect of eyelashes on attractiveness in a similar manner, regardless of the ethnicity of the participants or the stimuli. While there are sex differences in perception of facial attractiveness perception at neural and behavioral levels [^18,29^; cf.^30^], the convergence across the sexes in the present study speak to the robustness of the present effect. This point is supported further by the fact that we obtained the same inverted-U pattern whether participants were asked to rank the attractiveness of the faces against one another as in Ref.^1^ or to numerically rate each face's attractiveness individually as in the present study. In sum, the inverted-U function generalizes across tasks, populations, and stimulus types.

The current study is not without limitations. While using the 3D-generated stimuli instead of actual faces decreased the ecological validity of this study, on the other hand, it provided the possibility to control for other aspects of the stimuli, such as hair color and style, facial ratios, stimuli exposure and brightness, etc. To increase ecological validity, future research may choose to use actual faces for investigating the effect of eyelash length on attractiveness perception. Another limitation of the current study is the use of only female faces which constrains the generalizability of the effect. While Pazhoohi and Kingstone^1^ reported a similar inverted-U function for male faces—albeit skewed for shorter ratios—future research might choose to include male faces from different ethnicities. Moreover, using a single identity for generating different ethnicities is another limitation of the generalizability of this study, as one might argue that this specific pattern of results might have arisen from the particular combination of facial features.

The results of the current study, along with those of Pazhoohi and Kingstone^1^, are partially supportive of a previous study conducted by Adam^31^ on the attractiveness of the eyelash lengths. Specifically, Adam^31^ created eyelashes on stimuli as twice the length of the original version, as well as making them fuller and darker. Comparing the attractiveness of the faces with the manipulated eyelashes to the original ones, she found that longer eyelashes contribute to higher attractiveness ratings. The Adam’s study was limited in the ranges of the eyelash lengths, most specifically in the upper range, which is resolved by the current study as well as that of Pazhoohi and Kingstone^1^. While using a more diverse sample of faces (in terms of sex, ethnicity, and age) of real people smiling, might have resulted in more generalizability of Adam’s study^31^, it might have also left many confounding variables in the stimuli compared to the current study.

The hypothesis presented in this study was based on research revealing that very long and short eyelashes can indicate congenital and noncongenital diseases from health and genetic perspectives. We argued that attractiveness of eyelash length should be considered from an adaptive evolutionary viewpoint which posits that attractiveness is an honest indicator of health and genetic quality^3,4^. However, the current study was limited by only measuring the attractiveness of eyelash length. An outstanding and substantive question is whether health, broadly construed, is a critical factor, or even the critical factor. To address this systematically, future research might include whether and how perception of health interacts with attractiveness ratings in this regard. While Adam^31^ has provided preliminary evidence on this issue by showing that shorter eyelashes are considered less healthy, it is an open question as to whether health is the factor that is driving the inverted-U function we observed for attractiveness. Nonetheless, the findings of the current study add to the previous research that has shown a correspondence between attractiveness and fitness for other features such as waist to hip ratio^32^, hair length^33^, breasts^34^, and scleral colour^35^. Finally, the inverted-U shape function of attractiveness ratings might also suggest a preference for typicality where an optimum eyelash length matches (a) the typical or average length of the population and/or (b) the typical or average length experienced by individuals. Were one to garner reliable data on these two matters, one might tease apart the issue by finding, for example, that attractiveness ratings differ between ethnicities but measurements or typicality ratings do not; an outcome that would suggest that attractiveness ratings are not driven by typicality. An alternative approach would be to experimentally manipulate the base rate information on eyelash lengths in different populations and cross that with individuals being exposed to different frequencies of eyelash lengths. Again, if attractiveness ratings differ between ethnicities but measurements or typicality ratings do not, then the outcome would suggest that attractiveness ratings are not driven by typicality.

The current study has revealed that regardless of the sex or ethnicity of an observer, the effect of eyelash length on the perceived attractiveness of a women's face follows an inverted-U function, with attractiveness rising then falling with increasing eyelash length. Critically, this pattern is observed for depiction of faces of different ethnicities (and eye colour). Collectively, the data dovetail with the view that the optimum eyelash length evolved to maximum protect and facilitate vision. As such, deviations from that optimum are perceived to be less attractive as they may serve as signals of ill health. This effect is robust across different forms of task and measurement. Nevertheless, the effects are not absolute, with the preferred range varying at times with the ethnicity of the observer or the image.

### Supplementary Information

Supplementary Table S1.

## Data Availability

The data is available as supplementary material.
