# Supplementary material for: Eyelash length attractiveness across ethnicities
Source: Sci Rep. 2023 Sep 8;13:14849. doi: 10.1038/s41598-023-41739-5 (PMC10491613; doi:10.1038/s41598-023-41739-5)
Supplement: Supplementary file 1 — Supplementary Table S1. [file 41598_2023_41739_MOESM1_ESM.docx]

| Table S1. Results for Curve Estimation for Eyelash Length Predicting Attractiveness Ratings for Each Participant Ethnicity and Stimulus Ethnicity | | | | | | | | | |
| --- | --- | --- | --- | --- | --- | --- | --- | --- | --- |
| Participant Ethnicity | Stimulus Ethnicity |  | Unstandardized coefficients B | SE B | Standard coefficient β | *t* | *F* (df) | *p* | Adjusted R^2^ |
| Asian | Indian |  |  |  |  |  | 99.40 (2, 1097) | < .001 | 0.15 |
|  |  | Length | 0.92 | 0.07 | 1.73 | 14.10 |  |  |  |
|  |  | Length^2^ | -0.07 | 0.01 | -1.69 | -13.78 |  |  |  |
|  | Black |  |  |  |  |  | 57.09 (2, 1097) | < .001 | 0.09 |
|  |  | Length | 0.53 | 0.06 | 1.04 | 8.22 |  |  |  |
|  |  | Length^2^ | -0.03 | 0.01 | -0.82 | -6.46 |  |  |  |
|  | Asian |  |  |  |  |  | 101.41 (2, 1097) | < .001 | 0.15 |
|  |  | Length | 0.87 | 0.06 | 1.69 | 13.78 |  |  |  |
|  |  | Length^2^ | -0.07 | 0.01 | -1.54 | -12.60 |  |  |  |
|  | White |  |  |  |  |  | 104.42 (2, 1097) | < .001 | 0.16 |
|  |  | Length | 0.95 | 0.07 | 1.76 | 14.44 |  |  |  |
|  |  | Length^2^ | -0.07 | 0.01 | -1.70 | -13.92 |  |  |  |
| Black | Indian |  |  |  |  |  | 114.76 (2, 1086) | < .001 | 0.17 |
|  |  | Length | 0.88 | 0.07 | 1.66 | 13.66 |  |  |  |
|  |  | Length^2^ | -0.06 | 0.01 | -1.44 | -11.82 |  |  |  |
|  | Black |  |  |  |  |  | 68.97 (2, 1086) | < .001 | 0.11 |
|  |  | Length | 0.43 | 0.07 | 0.80 | 6.38 |  |  |  |
|  |  | Length^2^ | -0.02 | 0.01 | -0.50 | -3.98 |  |  |  |
|  | Asian |  |  |  |  |  | 138.37 (2, 1086) | < .001 | 0.20 |
|  |  | Length | 0.92 | 0.07 | 1.69 | 14.17 |  |  |  |
|  |  | Length^2^ | -0.06 | 0.01 | -1.41 | -11.82 |  |  |  |
|  | White |  |  |  |  |  | 108.67 (2, 1086) | < .001 | 0.16 |
|  |  | Length | 0.97 | 0.07 | 1.75 | 14.35 |  |  |  |
|  |  | Length^2^ | -0.07 | 0.01 | -1.61 | -13.21 |  |  |  |
| White | Indian |  |  |  |  |  | 111.01 (2, 1317) | < .001 | 0.14 |
|  |  | Length | 0.82 | 0.06 | 1.46 | 13.06 |  |  |  |
|  |  | Length^2^ | -0.05 | 0.01 | -1.24 | -11.09 |  |  |  |
|  | Black |  |  |  |  |  | 95.44 (2, 1317) | < .001 | 0.12 |
|  |  | Length | 0.45 | 0.06 | 0.83 | 7.36 |  |  |  |
|  |  | Length^2^ | -0.02 | 0.01 | -0.51 | -4.51 |  |  |  |
|  | Asian |  |  |  |  |  | 131.23 (2, 1317) | < .001 | 0.16 |
|  |  | Length | 0.86 | 0.60 | 1.56 | 14.04 |  |  |  |
|  |  | Length^2^ | -0.06 | 0.01 | -1.31 | 11.85 |  |  |  |
|  | White |  |  |  |  |  | 126.68 (2, 1317) | < .001 | 0.16 |
|  |  | Length | 0.99 | 0.06 | 1.75 | 15.78 |  |  |  |
|  |  | Length^2^ | -0.07 | 0.01 | -1.66 | -14.90 |  |  |  |
